# ASSOCIATION BETWEEN SOCIAL SUPPORT AND ANXIETY IN OLDER ADULTS NEW TO LONG-TERM SERVICES AND SUPPORTS

**DOI:** 10.1093/geroni/igz038.587

**Published:** 2019-11-08

**Authors:** Yeji Hwang, Nancy Hodgson, Justine Sefcik, Augustine Boateng, Anjali Rajpara, Kierra Foley, Karen Hirschman

**Affiliations:** University of Pennsylvania, Philadelphia, Pennsylvania, United States

## Abstract

Anxiety symptoms are common among older adults and are often associated with adverse outcomes. Thus, it is important to examine modifiable factors and manage anxiety symptoms in this population. While many biological and psychological factors related to anxiety symptoms in older adults have been found, little is known about social factors which are essential in one’s mental health. The purpose of this study was to examine the prevalence of anxiety symptoms among older adults new to long-term services and supports (LTSS) and to investigate the relationship between social support and presence of anxiety. This was a secondary data analysis from a study funded to examine health related quality of life in older adults new to LTSS. Anxiety was assessed using a single item, “Recently, how often have you felt anxious?” and the answers were dichotomized into “anxiety” (ratings: ‘very often’, ‘often’, ‘sometimes’, and ‘seldom’) and “no anxiety” (rating: ‘never’). Social support was measured by Medical Outcomes Study Social Support Scale. Prevalence of anxiety symptoms in this sample was 82.7% (n=225). In multivariate logistic regression, adjusting for age, gender, LTSS type, cognitive status, physical and emotional health, and depressive symptoms, older adults with more tangible social supports had lower odds of having anxiety symptoms (Odds ratio=0.515; 95% CI: 0.289-0.919, p=0.025). Improving access to tangible social supports for older adults at the start of LTSS may impact anxiety in older adults. Implications for future research and intervention development to provide tangible social support to older adults in LTSS will be discussed.

